# Role of the *Candida albicans MNN1* gene family in cell wall structure and virulence

**DOI:** 10.1186/1756-0500-6-294

**Published:** 2013-07-26

**Authors:** Steven Bates, Rebecca A Hall, Jill Cheetham, Mihai G Netea, Donna M MacCallum, Alistair JP Brown, Frank C Odds, Neil AR Gow

**Affiliations:** 1 College of Life and Environmental Sciences, University of Exeter, Exeter, EX4 4QD, UK; 2 School of Medical Sciences, University of Aberdeen, Aberdeen, AB25 2ZD, UK; 3 Department of Medicine, Radboud University Nijmegen Medical Center, Nijmegen, HB 6500, The Netherlands; 4 Current address: School of Biosciences and Institute of Microbiology & Infection, The University of Birmingham, Edgbaston, Birmingham, B15 2TT, UK

**Keywords:** *Candida albicans*, Glycosylation, Mannoproteins, Cell wall, *MNN1*, Virulence

## Abstract

**Background:**

The *Candida albicans* cell wall is the first point of contact with the host, and its outer surface is heavily enriched in mannoproteins modified through the addition of *N*- and *O*-mannan. Previous work, using mutants with gross defects in glycosylation, has clearly identified the importance of mannan in the host-pathogen interaction, immune recognition and virulence. Here we report the first analysis of the *MNN1* gene family, which contains six members predicted to act as α-1,3 mannosyltransferases in the terminal stages of glycosylation.

**Findings:**

We generated single null mutants in all members of the *C. albicans MNN1* gene family, and disruption of *MNN14* led to both *in vitr*o and *in vivo* defects. Null mutants in other members of the family demonstrated no phenotypic defects, suggesting that these members may display functional redundancy. The *mnn14*Δ null mutant displayed hypersensitivity to agents associated with cell wall and glycosylation defects, suggesting an altered cell wall structure. However, no gross changes in cell wall composition or *N*-glycosylation were identified in this mutant, although an extension of phosphomannan chain length was apparent. Although the cell wall defects associated with the *mnn14*Δ mutant were subtle, this mutant displayed a severe attenuation of virulence in a murine infection model.

**Conclusion:**

Mnn14 plays a distinct role from other members of the *MNN1* family, demonstrating that specific *N*-glycan outer chain epitopes are required in the host-pathogen interaction and virulence.

## Findings

### Background

*Candida albicans* is the most common opportunistic fungal pathogen of humans causing superficial infections of the mucosa, and life threatening systemic infections in immunocompromised and severely ill patients [1-3]. The fungal cell wall is a dynamic structure required for maintaining cell shape and providing protection from changes in the extracellular environment. In addition, the cell wall acts as the first point of contact with the host and plays an essential role in the host-fungal interaction. The cell wall is composed of an inner skeletal layer of β-glucans and chitin, decorated with an outer layer enriched in mannoproteins that are heavily post-translationally modified through the addition of *N*- and *O*-linked mannans [4-6]. These mannans have been shown to play a vital role in cell wall integrity, adhesion and virulence, and constitute one of the main *C. albicans* pathogen associated molecular patterns (PAMPs) recognised by the host innate immune system [7-16].

In *C. albicans* structural studies of *O*-mannan have shown that it typically consists of one to five α1,2-linked mannose residues attached to serine or threonine, and that these are required for full virulence [11,12]. In addition studies using anti-β-mannan specific antibodies have demonstrated that *O*-mannan may also contain β1,2-linked mannose residues [13], presumably added by members of the *BMT* family [14]. This is different to *Saccharomyces cerevisiae* where *O*-mannan consists of one to two α1,2-linked mannose residues capped with α1,3-linked mannose residues transferred by members of the Mnn1 family. *N*-glycosylation is initiated in the endoplasmic reticulum (ER) with the transfer of the *N*-mannan precursor (Glc_3_Man_9_GlcNAc_2_) to specific asparagine residues in the protein. The precursor is then processed by ER resident glycosidases to form the mature Man_8_GlcNAc_2_ core conserved across eukaryotes [10]. In *C. albicans* as well as other fungi, the core is then extensively modified through outer chain elaboration as the protein passes through the Golgi [7,14,16,17]. Outer chain elaboration is initiated through the addition of a single α1,6-linked mannose residue to the core by Och1 [7], and the subsequent extension of the α1,6-backbone by the sequential action of the mannan polymerase I and II enzyme complexes [17,18]. The α1,6-backbone is then further elaborated with side chains, which in *C. albicans* consists of α1,2-, α1,3-, and β1,2-linked mannose residues [14,16,19]. In addition both *N*-mannan and *O*-mannan structures can be further modified through the addition of phosphomannan, which in *C. albicans* consists of a chain of β1,2-linked mannose residues attached through a phosphodiester linkage [10,20,21].

Previous work has clearly identified outer chain *N*-glycosylation as an important factor in the host-fungal interaction and virulence [7,8,10,14-16]. However, most work to date has focused on the early stages of outer chain elaboration where the mutants display gross defects in glycosylation. In *S. cerevisiae* the *MNN1/2* family contains genes involved in the elaboration of *O*- and *N*-mannan; in particular the *MNN1* sub-family encodes α1,3-mannosyltranferases important for the addition of the terminal mannose residues in *O*- and *N*-mannan [22,23]. In *C. albicans* we have identified six *MNN1* family members and here we report the first analysis of this gene family in *C. albicans*. Null mutants in most of its members displayed no alteration of phenotype, suggesting possible functional redundancy. However, the *mnn14*Δ mutant, whilst displaying only subtle cell wall changes, was severely attenuated in virulence, demonstrating that specific glycans are important in the host-fungal interaction.

## Results and discussion

### Analysis and disruption of *C. albicans MNN1* family members

**Figure 1:**
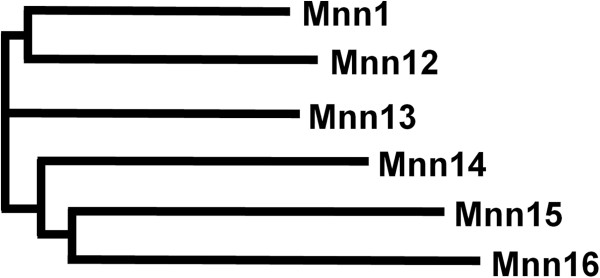
**Phylogram of the *C. albicans MNN1 *gene family.** A multiple sequence alignment and phylogram of the six *MNN1* orthologues was generated in Clustal Omega (version 1.1.0).

Our analysis of assembly 21 of the *C. albicans* genome identified a gene family of 12 members homologous to the *S. cerevisiae MNN1/2* family. Similar to *S. cerevisiae* this family could be divided into two subfamilies based on homology to either *MNN1* or *MNN2*. Six members of the *C. albicans MNN1* family were identified (orf19.4279, orf19.4900, orf19.4270, orf19.6996, orf19.753 and orf19.6313) and designated *MNN1*, *MNN12*, *MNN13*, *MNN14*, *MNN15* and *MNN16*. The proteins encoded by this family share 19-44% homology between members (Table 1, Figure 1) and a characteristic type II membrane protein topology, with a single N-terminal membrane spanning region (17–19 amino acids) preceded by a short (5–13 amino acid) cytosolic tail. All Mnn1 family members, except Mnn16, contain the conserved DxD motif known to be important for substrate and co-factor binding [24], suggesting that Mnn16 may have altered functionality. The *MNN1* family shows varying numbers in the sequenced CUG clade species [25] with *C. albicans*, *Candida tropicalis* and *Candida parapsilosis* encoding 6, 8, and 4 members respectively, compared to the non-pathogenic or infrequent pathogens, *Lodderomyces elongisporus*, *Candida guilliermondii*, *Candida lusitaniae*, and *Debaryomyces hansenii* which have 5, 4, 3 and 3 members respectively.

**Table 1:** **Homology of *MNN1 *gene family products**

**Protein**	**% Identity (% similarity)**^**a**^					
	**Mnn1**	**Mnn12**	**Mnn13**	**Mnn14**	**Mnn15**	**Mnn16**
Mnn1	100%	-	-	-	-	-
	(100%)					
Mnn12	43.8%	100%	-	-	-	-
	(60.7%)	(100%)				
Mnn13	42.8%	37.7%	100%	-	-	-
	(62.0%)	(55.9%)	(100%)			
Mnn14	31.3%	32.9%	32.2%	100%	-	-
	(47.3%)	(49.34%)	(49.1%)	(100%)		
Mnn15	24.3%	24.8%	25.9%	26.1%	100%	-
	(39.5%)	(39.6%)	(41.8%)	(42.8%)	(100%)	
Mnn16	21.9%	19.0%	21.2%	24.6%	21.4%	100%
	(35.4%)	(31.8%)	(36.1%)	(39.8%)	(36.5%)	(100%)

^a^ Identity and similarity were calculated over the full length of the protein.

**Figure 2:**
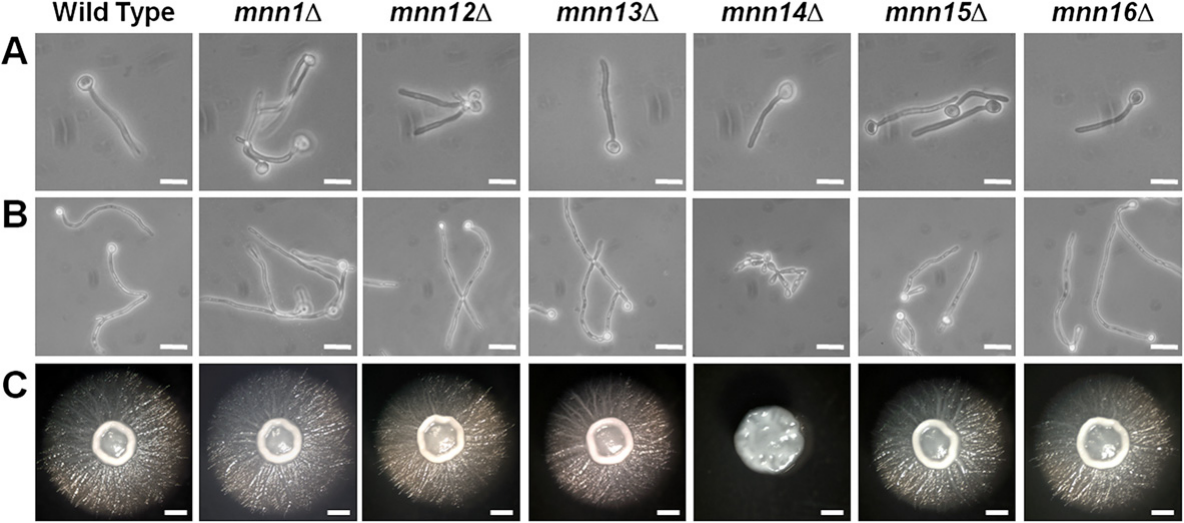
**Cell and colony morphology of *MNN1 *family mutants.** Cell morphology after growth at 37°C in 20% serum for 4 h **(A)** and at 37°C in Lee’s at pH6.5 for 6 h **(B)**. Scale bar, 10 μm. **(C)** Colony morphology after 6 days growth at 30°C on solid Spider medium. Scale bar, 1 mm.

Single null mutants in all *MNN1* family members were generated through sequential gene deletion, and the *URA3* marker was introduced at the *RPS1* locus to avoid problems associated with its ectopic expression. To control against second site mutations two independently constructed null mutants were generated for each member of the family and shown to display comparable phenotypes. The mutants all had growth rates similar to the wild type control strain (doubling time 1.4 h) in SC medium at 30°C, and displayed no morphological defects, such as cellular aggregation, which has previously been seen in mutants displaying glycosylation defects [7-10,16]. In terms of hyphal development all of the *MNN1* family mutants underwent morphogenesis in response to 20% serum. However, the *mnn14*Δ null mutant displayed a defect in response to the weaker hypha-inducing signal of pH and temperature, with cells constitutively forming pseudohyphae in response to Lee’s medium at pH 6.5 and 37°C. In addition, the *mnn14*Δ null mutant also failed to form filaments on solid spider medium (Figure 2).

### Glycosylation defects in the *MNN1* family mutants

**Figure 3:**
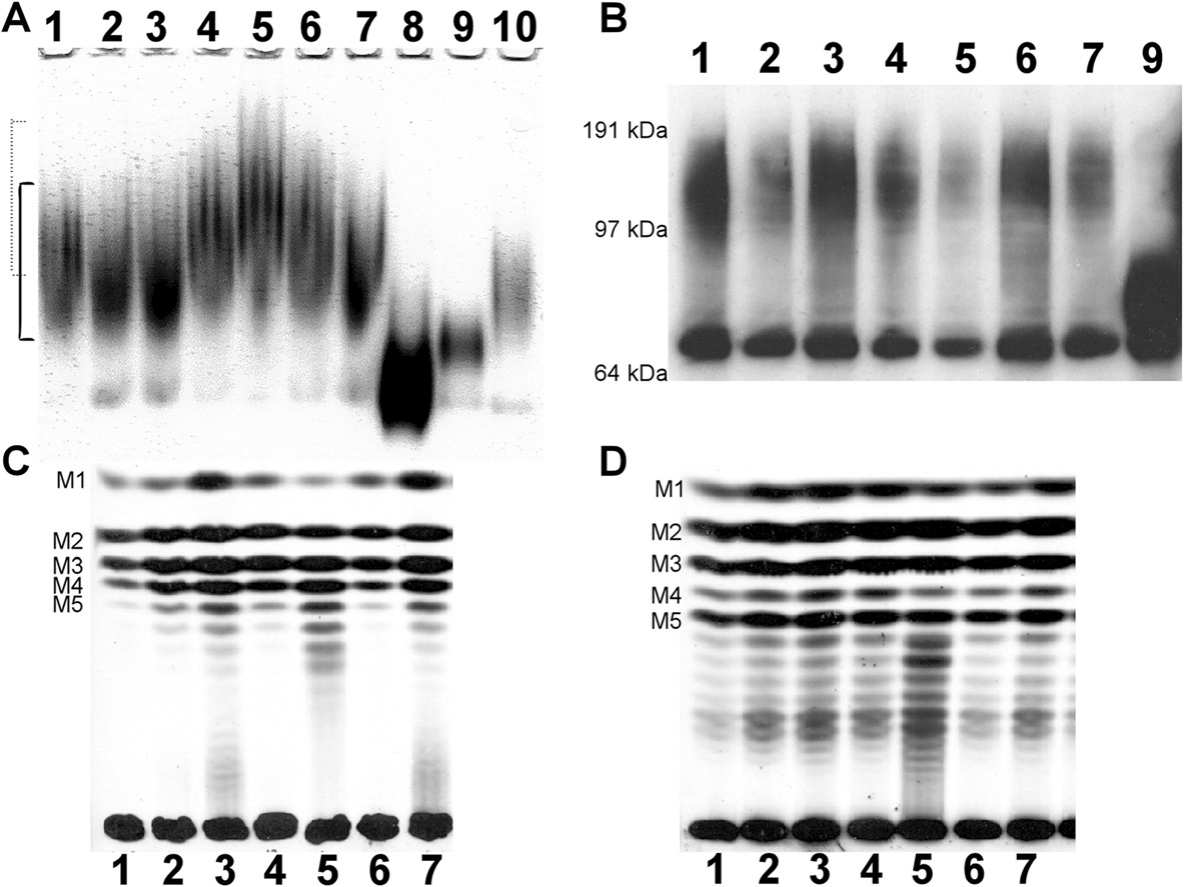
**Glycosylation defects in *C. albicans MNN1 *family null mutants.** The extent of *N*-glycosylation was determined by activity staining of Hex1 (β-*N*-acetylhexosaminidase) after protein samples were separated by non-denaturing gel electrophoresis **(*A*)**. The continuous and dotted lines indicate the normal and decreased electrophoretic mobility of Hex1 respectively. The degree of *N*-glycosylation modification was also determined by western blot analysis of Hex1-V5-6× His tagged strains **(*B*)**. Hex1-V5-6× His is apparent in both an unmodified (67 kDa) and a heavily glycosylated (~125 kDa) form. Acid-labile phosphomannan **(*C*)** and β-eliminated *O*-mannan **(*D*)** were released from [2-^3^H] mannose-labelled cells and separated by TLC. Samples are as follows: *lane 1*, wild type; *lane 2*, *mnn1∆*; *lane 3*, *mnn12∆*; *lane 4*, *mnn13∆*; *lane 5*, *mnn14∆*; *lane 6*, *mnn15∆*; *lane 7*, *mnn16∆*; *lane 8*, *och1∆*; *lane 9*, *pmr1∆*; *lane 10*, *mnt1∆-mnt2∆*.

The effect of disruption of the *MNN1* family members on *N*-linked glycosylation was initially assessed through monitoring the electrophoretic mobility of secreted Hex1 (β-*N*-acetylhexosaminidase) by activity staining following native gel electrophoresis [7]. None of the *MNN1* family mutants displayed the increased electrophoretic mobility of Hex1 that is normally associated with a deficiency in *N*-glycosylation, as demonstrated by *och1*Δ and *pmr1*Δ mutants [7,8], indicating no gross *N*-glycosylation defects in the *MNN1* family mutants (Figure 3*A*). However, the *mnn14*Δ null mutant instead demonstrated a noticeable decrease in Hex1 mobility following native gel electrophoresis (Figure 3*A*). This decrease in electrophoretic mobility could be suggestive of an increased level of glycosylation of Hex1 in this mutant. Alternatively, an alteration in the level, or extent of, phosphomannan modification could affect the surface charge of Hex1, which would impact on its mobility in a native gel.

To screen for more subtle glycosylation defects, and to discount the impact of altered surface charge on electrophoretic mobility, we epitope tagged Hex1 with V5-6xHis in the null mutants and conducted Western blot analysis of Hex1-V5-6xHis following standard denaturing gel electrophoresis. Following Western blot analysis Hex1-V5-6xHis was apparent in both an unmodified (67 kDa) and a heavily glycosylated (~125 kDa) form in soluble protein extracts. In the *pmr1*Δ mutant, which is known to display glycosylation defects [8], the glycosylated form of Hex1-V5-6xHis demonstrated a clear increase in electrophoretic mobility characteristic of this mutant’s gross defect in glycosylation. However, no change in electrophoretic mobility of the glycosylated form of Hex1-V5-6xHis was observed for any of the *MNN1* family mutants (Figure 3*B*), demonstrating that none of these mutants displayed a gross defect in *N*-glycosylation. In addition, this would suggest that the decreased electrophoretic mobility of Hex1 seen in the *mnn14*Δ null mutant following native gel electrophoresis and activity staining is likely to be due to differences in the proteins surface charge and not the overall level of glycosylation.

Phosphomannan, attached to *O*- and *N*- mannan through a phosphodiester linkage, can affect the native charge of proteins. The overall level of phosphomannan incorporation into the cell wall can be estimated through the use of the phthalocyanine dye, Alcian Blue, which binds to phosphorylated polysaccharides [20,26]. None of the *MNN1* family null mutants displayed altered Alcian Blue binding (Table 2), indicating that each displays a similar level of phosphomannan at the cell surface. This would also suggest that the decreased native gel mobility of Hex1 from *mnn14*Δ cells was not due to a decrease in the overall level of phosphomannan present. The surface charge associated with phosphomannan is also dependent on the chain length of β1,2-mannose residues present [26,27]. Therefore, we also directly analysed phosphomannan structure in the *MNN1* family null mutants by TLC. The wild type strain, and the majority of the mutants, displayed the expected profile of one to five β1,2-mannose residues. However, the *mnn14*Δ null mutant clearly displayed an altered profile with a preponderance for longer chains and an increased chain length of one to eight residues (Figure 3*C*). Extension of the phosphomannan chain has previously been identified as playing a major role in the modulation of cell surface charge, with longer phosphomannan chains resulting in the loss of charge demonstrated by an increase in hydrophobicity of the cell wall [26,27]. Therefore the increase in the degree of phosphomannan extension seen in the *mnn14*Δ null mutant could reduce the surface charge associated with Hex1 which would then explain its decreased mobility under native conditions even though its overall level of modification is not grossly alerted.

**Table 2:** Alcian blue binding

**Strain genotype**	**Alcian blue binding ± SD (μg bound/OD**_**600 **_**1.0)**
Wild type	183.1 ± 8.7
*mnn1Δ*	148.2 ± 21.6
*mnn12Δ*	143.3 ± 37.4
*mnn13Δ*	175.3 ± 7.4
*mnn14Δ*	181.9 ± 3.9
*mnn15Δ*	184.1 ± 2.6
*mnn16Δ*	191.1 ± 6.7
*och1Δ*	25.2 ± 2.2

The structure of *O*-linked mannans was also assessed by TLC and no significant differences were observed in any of the *MNN1* family mutants (Figure 3*D*). This is consistent with the previous finding that Mnt1 and Mnt2 are the principal enzymes involved in the extension of *O*-glycans, and that *C. albicans* lacks α1,3-linked residues in its *O*-mannan [11].

### Cell wall associated defects

**Figure 4:**
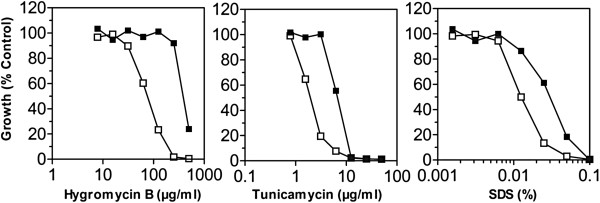
**Sensitivity of *C. albicans mnn14Δ *null mutant to cell wall perturbing agents.** The sensitivities of the wild type (*closed squares*) and *Camnn14Δ* null mutant (*open squares*) to cell wall perturbing agents were determined by a broth microdilution method. The agents to which the *Camnn14Δ* null mutant displayed hypersensitivity are shown (hygromycin B, tunicamycin, and SDS).

To examine cell wall integrity we screened the *MNN1* family null mutants for sensitivity to a range of cell wall perturbing agents. The *mnn14*Δ null mutant was clearly hypersensitive to SDS, tunicamycin, and hygromycin B which is characteristic of glycosylation mutants (Figure 4). However, no increase in sensitivity was seen to the cell wall perturbing agent Calcofluor White, suggesting that the *mnn14*Δ null mutant does not have a defect in the general robustness of the cell wall. No other family members displayed altered sensitivity to any of the compounds tested. Changes in cell wall structure were also assessed through HPLC sugar composition analysis, but no gross alteration in the relative proportions of the three main cell wall polysaccharides (glucan, mannan and chitin) was identified for any of the *MNN1* family null mutants (data not shown).

**Figure 5:**
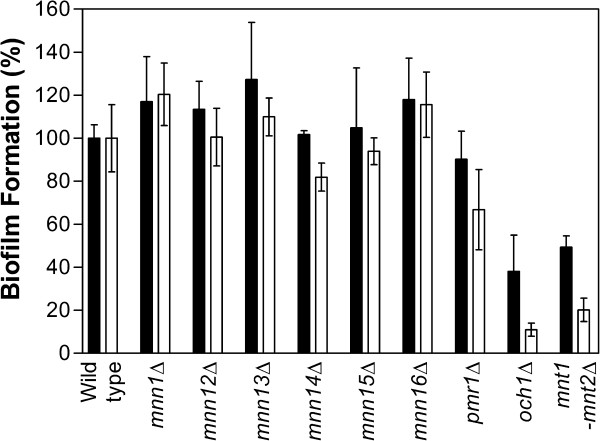
**Biofilm formation of *MNN1 *family mutants.** Biofilm formation was assessed in 96 well plates using a tetrazolium salt (XTT) reduction assay following growth in SC medium for 24 h (*closed bars*) and 48 h (*open bars*).

Defects in the initiation of *O*-glycosylation or treatment with tunicamycin, which inhibits *N*-glycosylation, have previously been shown to impair biofilm formation [28,29]. We therefore screened the *MNN1* family mutants for defects in biofilm formation, using a 96 well microplate model and XTT reduction assay to measure metabolic activity of the cells within the biofilm. As predicted the *och1∆* mutant [7], which has a gross defect in *N*-linked glycosylation, displayed a severe biofilm defect demonstrating a 90% reduction in biofilm formation at 48 h, therefore confirming the importance of *N*-mannan in biofilm formation. In addition the *mnt1*Δ*-mnt2*Δ double mutant [11], which lacks the 4 terminal mannose residues in *O*-mannan, also displayed a clear biofilm defect (80% reduction). This indicates that the extension of *O*-mannan, in addition to the initiation of its synthesis, is also important for biofilm formation. However, none of the *MNN1* family single mutants displayed a significant defect in biofilm formation (Figure 5). This lack of a phenotypic defect may be due to functional redundancy in the *MNN1* family. Alternatively, it may suggest that only gross *N*-glycosylation defects impact upon biofilm formation rather than its being dependent on specific epitopes. Indeed, the *pmr1*Δ null mutant, which displays both *O*- and *N*-linked glycosylation defects, but to a lesser extent than the individual defects seen in the *och1*Δ or *mnt1*Δ*-mnt2*Δ mutants, only displays a subtle biofilm defect (40% reduction at 48 h). Therefore, biofilm formation is dependent both on correct *O*-glycosylation and the overall level of *N*-glycosylation, presumably through their impact on cellular interactions.

### Host-pathogen interaction and virulence

As cell wall mannan has been shown to play an important role in the stimulation of the host response [15] we tested the ability of the null mutants to induce cytokine production by human PBMCs. All the *MNN1* family mutants were as potent at stimulating TNFα and IL-6 production as wild type *C. albicans* (data not shown). Virulence of the single mutants was tested in a *Galleria mellonella* larvae model of infection. In this model all larvae infected with the wild type strain succumbed to infection by day 2, whereas 75% of larvae infected with the *pmr1*Δ mutant, which displays a gross defect in glycosylation, survived to the end of the experiment. However, none of the *MNN1* family mutants displayed a virulence defect, demonstrating that none of the individual family members are required for virulence in this model. Because the *mnn14*Δ mutant displayed an altered phenotype *in vitro* we tested its virulence in a murine model of disseminated infection; in addition the *mnn1*Δ and *mnn12*Δ mutants were also tested. The *mnn1*Δ and *mnn12*Δ null mutants were unaltered in virulence. However, the *mnn14*Δ null mutant was clearly attenuated in virulence (log-rank test; p < 0.001) with all mice surviving to the end of the experiment at 28 days compared to a median survival time of 9 days for the wild type control. Mice infected with the *mnn14*Δ null mutant also displayed a clear reduction in tissue burdens at 28 days compared to that seen post-mortem following infection with the wild type strain, with a >2 log reduction in kidney burdens (log_10_ CFU/g 4.0 ± 1.3 cf. 6.5 ± 0.4) and a 1 log reduction in the brain (log_10_ CFU/g 3.3 ± 0.9 cf. 4.3 ± 0.4). Hence the *mnn14*Δ null mutant was significantly attenuated in virulence in the murine model of systemic candidiasis.

## Conclusion

The *C. albicans* cell wall is the immediate point of contact between the invading fungus and the host, and previous work has clearly identified both *O*- and *N*- mannan structures as important in the host-pathogen interaction and virulence. In this study, we present the first analysis of the *C. albicans MNN1* gene family, which is predicted to encode capping enzymes involved in the terminal stages of glycosylation. We generated single null mutants in the six members of the *C. albicans MNN1* gene family. The majority of the mutants generated, with the exception of the *mnn14*Δ null mutant, demonstrated no discernible change in phenotype potentially due to functional redundancy as seen with some other *C. albicans* mannosyltransferase gene families [9,11,16]. Disruption of *MNN14* however led to both *in vitro* and *in vivo* defects. The *mnn14*Δ null mutant displayed subtle morphogenesis defects and hypersensitivity to agents associated with cell wall and glycosylation defects, suggesting altered cell wall structure or permeability. However, no gross changes in cell wall composition or *N*-glycan extension were identified, although an extension of phosphomannan chains was apparent. This extension of phosphomannan in the *mnn14*Δ null mutant could be a compensatory mechanism for its absence, through an increase in the expression, or activity, of other mannosyltransferases. Alternatively Mnn14 could potentially act as a capping enzyme to terminate extension similar to the proposed role of *MNN1* in *S. cerevisiae*[30]. Although the *mnn14*Δ null mutant only displayed subtle cell wall defects this mutant did display a severe defect in virulence in a murine infection model, apparent both in terms of overall survival and associated tissue burdens. This virulence defect was not associated with differences in the induction of inflammatory responses, as no defect was seen in the TNFα and IL-6 host cytokine production *in vitro*, which suggests that the virulence defect may not be due to an alteration of the host response. Overall, therefore, the *MNN1* family appears to display redundancy, with the exception of *MNN14,* which may therefore play a distinct role either in the synthesis of specific epitopes or in the modification of a discrete protein(s) required for virulence and the host-pathogen interaction.

## Methods

### Strains, media and culture conditions

*C. albicans* strains were grown in yeast and hyphal forms as described previously [7]. To induce β-*N*-acetylhexosaminidase expression strains were grown in SC-GlcNAc (0.67% yeast nitrogen base, 0.079% complete supplement mixture, 25 mM *N*-acetylglucosamine). Biofilm formation in the mutants was assessed using a tetrazolium salt (XTT) reduction assay following growth in SC medium for 24 and 48 h [31]. Null mutants in *MNN1* (orf19.4279) and *MNN12* (orf19.4900) were generated by the “Ura-blaster” protocol in strain CAI-4 [32]. Briefly, the 5′ and 3′ regions of homology were amplified by PCR (MNN1 5′ primer pair MNN1-5H/5S, 3′ primer pair MNN1-3A/3B; MNN12 5′ primer pair MNN12-5H/5S, 3′ primer pair MNN12-3A/3B) and cloned into the HindIII/SphI and Asp718/BanII sites of the disruption vector pMB-7 respectively. The ura-blaster cassettes were then released by digestion with HindIII and BanII and genes disrupted in CAI-4 by sequential gene replacement and recycling of the *URA3* marker by selection on SD medium plus 5-fluoroorotic acid (1 mg/ml) and uridine (50 μg/ml). Single null mutants in the other family members, *MNN13* (orf19.4270), *MNN14* (orf19.6996) *MNN15* (orf19.753) and *MNN16* (orf19.6313), were generated through PCR mediated disruption [33]. Primers (MNNxx-KO-F/-R) were designed to contain 70 bp homology to the gene of interest, and disruption cassettes amplified from pSN40 (*C. maltosa LEU2*) and pSN52 (*C. dubliniensis HIS1*). Amplified cassettes were then sequentially transformed into strain SN78 to generate null mutants. The *URA3* marker was integrated at the *RPS1* locus in all mutants by transformation with the StuI-digested CIp10 vector [34], to avoid problems associated with its expression [35]. To control against second site mutations two independently constructed null mutants were generated and screened for each member of the family. Strain genotypes and the oligonucleotides used in their construction are listed in Tables 3 and 4. The wild type strain used for phenotypic studies throughout was NGY152 [35] derived from CAI-4 with the CIp10 vector integrated at the *RPS1* locus.

**Table 3:** ***C. albicans *strains**

**Strain**	**Parent strain**	**Genotype**	**Reference**
CAI-4	-	*ura3Δ::imm434/ura3*Δ::*imm434*	[32]
SN78	-	*ura3Δ*::*imm434*/*ura3Δ*::*imm434*, *leu2Δ*/*leu2Δ*, *his1Δ*/*his1Δ*	[33]
NGY152	CAI-4	As CAI-4 but *RPS1*/*rps1Δ*::CIp10	[35]
SBC106	CAI-4	As CAI-4 but *MNN1*/*mnn1Δ*::*hisG-URA3-hisG*	This study
SBC107	SBC106	As CAI-4 but *MNN1*/*mnn1Δ*::*hisG*	This study
SBC108	SBC107	As CAI-4 but *mnn1Δ*::*hisG*/*mnn1Δ*::*hisG-URA3-hisG*	This study
SBC109	CAI-4	As CAI-4 but *MNN12*/*mnn12Δ*::*hisG-URA3-hisG*	This study
SBC110	SBC109	As CAI-4 but *MNN12*/*mnn12Δ*::*hisG*	This study
SBC111	SBC110	As CAI-4 but *mnn12Δ*::*hisG*/*mnn12Δ*::*hisG-URA3-hisG*	This study
SBC112	SBC108	As CAI-4 but *mnn1Δ*::*hisG*/*mnn1Δ*::*hisG*	This study
SBC113	SBC111	As CAI-4 but *mnn12Δ*::*hisG*/*mnn12Δ*::*hisG*	This study
SBC114	SBC112	As CAI-4 but *mnn1Δ*::*hisG*/*mnn1Δ*::*hisG, RPS1*/*rps1Δ*::CIp10	This study
SBC115	SBC113	As CAI-4 but *mnn12Δ*::*hisG*/*mnn12Δ*::*hisG, RPS1*/*rps1Δ*::CIp10	This study
SBC122	SN78	As SN78 but *MNN13*/*mnn13Δ*::*CmLeu2*	This study
SBC123	SN78	As SN78 but *MNN14*/*mnn14Δ*::*CmLeu2*	This study
SBC124	SN78	As SN78 but *MNN15*/*mnn15Δ*::*CmLeu2*	This study
SBC135	SN78	As SN78 but *MNN16*/*mnn16Δ*::*CmLeu2*	This study
SBC126	SBC122	As SN78 but *mnn13Δ*::*CmLeu2/mnn13Δ::CdHIS1*	This study
SBC127	SBC123	As SN78 but *mnn14Δ*::*CmLeu2/mnn14Δ::CdHIS1*	This study
SBC128	SBC124	As SN78 but *mnn15Δ*::*CmLeu2/mnn15Δ::CdHIS1*	This study
SBC129	SBC135	As SN78 but *mnn16Δ*::*CmLeu2/mnn16Δ::CdHIS1*	This study
SBC130	SBC126	As SN78 but *mnn13Δ*::*CmLeu2/mnn13Δ::CdHIS1, RPS1/rps1Δ*::CIp10	This study
SBC131	SBC127	As SN78 but *mnn14Δ*::*CmLeu2/mnn14Δ::CdHIS1, RPS1/rps1Δ*::CIp10	This study
SBC132	SBC128	As SN78 but *mnn15Δ*::*CmLeu2/mnn15Δ::CdHIS1, RPS1/rps1Δ*::CIp10	This study
SBC133	SBC129	As SN78 but *mnn16Δ*::*CmLeu2/mnn16Δ::CdHIS1, RPS1/rps1Δ*::CIp10	This study
SBC163	SBC130	As SN78 but *mnn13Δ*::*CmLeu2/mnn13Δ::CdHIS1, RPS1/rps1Δ*::CIp10, *HEX1/HEX1-V5-6xHis-NAT1*	This study
SBC164	SBC131	As SN78 but *mnn14Δ*::*CmLeu2/mnn14Δ::CdHIS1, RPS1/rps1Δ*::CIp10, *HEX1/HEX1-V5-6xHis-NAT1*	This study
SBC165	SBC132	As SN78 but *mnn15Δ*::*CmLeu2/mnn15Δ::CdHIS1, RPS1/rps1Δ*::CIp10, *HEX1/HEX1-V5-6xHis-NAT1*	This study
SBC166	SBC133	As SN78 but *mnn16Δ*::*CmLeu2/mnn16Δ::CdHIS1, RPS1/rps1Δ*::CIp10, *HEX1/HEX1*-*V5*-*6xHis*-*NAT1*	This study
SBC161	SBC114	As CAI-4 but *mnn1*Δ::*hisG*/*mnn1*Δ::*hisG*, *RPS1*/*rps1*Δ::CIp10, *HEX1*/*HEX1*-*V5*-*6xHis*-*NAT1*	This study
SBC162	SBC115	As CAI-4 but *mnn12*Δ::*hisG*/*mnn12*Δ::*hisG*, *RPS1*/*rps1*Δ::CIp10, *HEX1*/*HEX1*-*V5*-*6xHis*-*NAT1*	This study
SBC158	NGY152	As NGY152 but *HEX1*/*HEX1*-*V5*-*6xHis*-*NAT1*	[36]
SBC159	-	*pmr1*Δ::*hisG*/*pmr1*Δ::*hisG HEX1*/*HEX1*-*V5*-*6xHis*-*URA3*	[36]
NGY355	-	As CAI-4 but *pmr1*Δ::*hisG*/*pmr1*Δ::*hisG*, *RPS1*/*rps1*Δ::CIp10	[8]
NGY357		As CAI-4 but *och1*Δ::*hisG*/*och1*Δ::*hisG*, *RPS1*/*rps1*Δ::CIp10	[7]
NGY337		As CAI-4 but *mnt1*-*mnt2*Δ::*hisG*/*mnt1*-*mnt2*Δ::*hisG*, *RPS1*/*rps1*Δ ::CIp10	[11]

**Table 4:** Oligonucleotides used for strain construction

**Primer**	**Sequence (5**′**-3**′**) **^**a**^
MNN1-5H	AAGCTTtctacagtggctgttaataatg
MNN1-5S	GCATGCgaaaactcatacaattcactaag
MNN1-3A	GGTACCtgcttactcaagtgttggtg
MNN1-3B	GAGCTCgttagtggcttgtatcaaagg
MNN12-5H	AAGCTTatcataacacgttcgttcagc
MNN12-5S	GCATGCtgacagatcatcgtcttcatc
MNN12-3A	GGTACCatgccattattccaccaatgg
MNN12-3B	GAGCTCgttgtgactgtggctccac
MNN13-KO-F	gttgactttgaagagccactgtatactttccctaagacggtgctatttgaaaacaacaacaatttggatgatttattatGCTCGGATCCACTAGTAACG
MNN13-KO-R	ttaatattttccaacccatacatctcctaagtaattgaaatatgcaatttcttcaggggtatactcaaccaataaaccaCCAGTGTGATGGATATCTGC
MNN14-KO-F	atgcattttatttggcaatcacaagccaattctggattaatactaaaaaatgaattatcactgacatcaagtatcccccGCTCGGATCCACTAGTAACG
MNN14-KO-R	ctataaataattatatcccctcatccatatctccccaagctttttaaaatgttctatttcctctggtgaataattaattCCAGTGTGATGGATATCTGC
MNN15-KO-F	cgtataagggaacttttacatgatagtgaaaaatttgatctcgagagtctaaagcaagctagcttggagataagaaaagGCTCGGATCCACTAGTAACG
MNN15-KO-R	ctttatccgaaccagttccaagtttctcagtttctaatttaacttttggcttcattttggatccaccagtgtgccatatCCAGTGTGATGGATATCTGC
MNN16-KO-F	gaagttccatcttaaaagatatgtcatagttacttcaatactactatcttttttcttattattcagacgacaatttcttGCTCGGATCCACTAGTAACG
MNN16-KO-R	ttaatcttgtaaccaaatttcaattaatttcttatattttttttgtaaactttgttcaacattaatagataatcctctaCCAGTGTGATGGATATCTGC

^a^ Gene-specific sequences are lowercase.

### Cell wall and glycosylation analysis

Null mutants were screened for sensitivity to cell wall stressing agents by the microdilution method as previously reported [7]. Briefly, standardised inocula (A_600_ = 0.01) of strains in YEPD medium were incubated with cell wall stressing agents, across a range of doubling dilutions, for 16 h at 30°C and the A_600_ was determined. The agents tested (maximum concentrations in parentheses) included Calcofluor White (500 μg/ml), Congo Red (500 μg/ml), SDS (0.1%), hygromycin B (500 μg/ml), NaCl (2 M) and tunicamycin (50 μg/ml). Phosphomannan content and *N*-glycosylation status were determined by Alcian Blue binding assays and β-*N*-acetylhexosaminidase (Hex1) native PAGE zymograms as reported elsewhere [7]. In addition the *N*-glycosylation status was assessed by Western blot analysis of mutant strains carrying the V5-6xHis tagged Hex1 protein as a glycosylation reporter as described previously [36]. Cell wall carbohydrate composition was determined by acid hydrolysis of the cell wall carbohydrate polymers and quantification of constituent monosaccharides following high performance anion exchange chromatography [37]. Finally, for the analysis of phosphomannan and *O*-linked mannan, strains were labelled with D-[2-^3^H] mannose, mannans released through mild acid treatment and β-elimination respectively and separated through thin layer chromatography (TLC) [7].

### Virulence assays

Isolation of human peripheral blood mononuclear cells (PMBCs) and cytokine stimulation assays were carried out as described previously [15]. For the wax moth larvae infection model groups of 8 larvae were infected with 3×10^5^ CFU of *C. albicans* through the last left proleg and survival monitored over 7 days at 37°C. To test virulence in a murine infection model groups of 6 female BALB/c mice were intravenously challenged with 3.6×10^4^ CFU/g (body weight) *C. albicans* and monitored over 28 days. Animals showing signs of illness or disease were humanely terminated and recorded as dying the following day, and those surviving the course of the experiment were terminated on day 28. The kidneys and brain were aseptically removed post-mortem, homogenised, and tissue burdens determined by viable counting. Experiments were conducted under the terms of a UK Home Office licence and were approved by the University of Aberdeen Ethical Review Committee.

## Competing interests

The authors declare that they have no competing interests.

## Authors’ contributions

SB conducted the experimental work and drafted the manuscript. RAH and JC assisted with the experimental work. MGN carried out the immunoassays. DMM conducted the murine infection model. SB, AJPB, FCO and NARG conceived the study, participated in its design and co-ordination. All authors read and approved the final manuscript.
